# Perioperative and Long-Term Outcomes of T-Shaped Esophagojejunostomy After Laparoscopic Total Gastrectomy for Gastric Cancer

**DOI:** 10.14740/jocmr6549

**Published:** 2026-05-31

**Authors:** Van-Huong Nguyen, Van-Chien Dinh, Van-Thuong Pham, Van-Quyet Ha, Ba-Thai Nguyen, Dinh-Hieu Nguyen, Minh-Tung Do

**Affiliations:** a Department of Gastrointestinal Surgery, Nghe An General Friendship Hospital, Nghe An, Vietnam; b Department of Surgery, Hai Phong University of Medicine and Pharmacy, Hai Phong, Vietnam; c Department of Surgery, Phenikaa University, Hanoi, Vietnam; d Department of Oncology, Nghe An General Friendship Hospital, Nghe An, Vietnam

**Keywords:** Stomach neoplasms, Gastrectomy, Laparoscopy, Esophagojejunostomy, Treatment Outcome

## Abstract

**Background:**

Intracorporeal esophagojejunostomy remains a technically challenging step in totally laparoscopic total gastrectomy (TLTG), and long-term outcome data for modified linear-stapled techniques are limited.

**Methods:**

We conducted an observational study including 167 consecutive patients who underwent TLTG with D2 lymphadenectomy and intracorporeal T-shaped functional end-to-end esophagojejunostomy between July 2017 and July 2025. Perioperative outcomes, postoperative complications, and long-term oncologic outcomes were analyzed.

**Results:**

All procedures were completed laparoscopically without conversion to open surgery. No anastomotic leakage occurred. Overall postoperative morbidity was 2.4%, and no postoperative mortality was observed. The mean follow-up duration was 44.1 ± 20.0 months, with complete survival follow-up. The 5-year overall survival rate was 41.8%.

**Conclusions:**

T-shaped functional end-to-end esophagojejunostomy after TLTG is safe and reproducible, with low anastomotic-related morbidity and acceptable long-term oncologic outcomes. These findings provide real-world evidence supporting standardized intracorporeal reconstruction following total gastrectomy.

## Introduction

Gastric cancer remains a major global health burden and is one of the leading causes of cancer-related mortality worldwide [1]. Total gastrectomy is the standard treatment for proximal and advanced gastric cancer, and surgical outcomes have improved with advances in perioperative management and minimally invasive techniques [2–4]. In particular, laparoscopic gastrectomy has been increasingly adopted due to its favorable short-term outcomes and comparable oncologic results to open surgery. As a result, totally laparoscopic total gastrectomy (TLTG) has become an attractive option in selected patients.

Despite these advances, intracorporeal esophagojejunostomy remains one of the most technically demanding steps in TLTG [5]. Various reconstruction techniques have been described, including the use of circular staplers and linear stapler-based methods such as overlap and functional end-to-end anastomosis [6, 7]. Each of these techniques has specific advantages but may also present limitations related to technical complexity, anastomotic configuration, or instrument handling in a confined laparoscopic field. Therefore, achieving a safe, reproducible, and technically straightforward intracorporeal anastomosis remains a key challenge.

To address these issues, we adopted a modified intracorporeal T-shaped esophagojejunostomy using linear staplers. This technique is constructed from an initial side-to-side anastomosis followed by a perpendicular stapling step that reshapes the anastomosis into a functional end-to-end configuration without a blind pouch. By simplifying the procedural steps and standardizing the anastomotic geometry, this approach may improve reproducibility in totally laparoscopic settings. In this study, we aimed to describe the surgical technique and evaluate its feasibility and clinical outcomes in patients undergoing TLTG.

## Materials and Methods

### Study design and setting

This observational study was conducted at tertiary referral hospitals in Viet Nam between July 2017 and July 2025. The study was designed to evaluate perioperative and long-term outcomes of a standardized intracorporeal esophagojejunostomy technique performed during TLTG for gastric cancer. The manuscript was prepared in accordance with the STROBE (Strengthening the Reporting of Observational Studies in Epidemiology) guidelines. Data were accessed for research purposes between August 15, 2025, and October 15, 2025.

### Ethical approval

The study protocol was reviewed and approved by the Institutional Review Board (IRB) of the participating institutions (IRB number: 01/CN-HDDD). Written informed consent was obtained from all patients prior to surgery. All procedures were performed in accordance with institutional ethical guidelines for research involving human participants and complied with the Declaration of Helsinki.

### Participants

Consecutive adult patients with histologically confirmed gastric adenocarcinoma who underwent elective TLTG with curative intent were enrolled. Eligibility criteria included tumors requiring total gastrectomy, absence of distant metastasis, and suitability for laparoscopic surgery based on preoperative assessment. Patients undergoing emergency surgery, palliative resection, conversion to open surgery, or those with incomplete clinical data were excluded.

### Surgical procedure

All procedures were performed by experienced gastric surgeons who had completed the learning curve for totally laparoscopic gastrectomy (>50 cases). Patients were placed in the supine split-leg position, and a standard five-port laparoscopic approach was used.

TLTG with D2 lymphadenectomy (LND) was performed according to the Japanese Gastric Cancer Association guidelines [8]. The left gastric vessels were ligated at their origin, and the duodenum was transected using a linear stapler. Proximal esophageal transection was performed above the esophagogastric junction, with frozen-section analysis routinely used to confirm negative margins.

**Figure 1:**
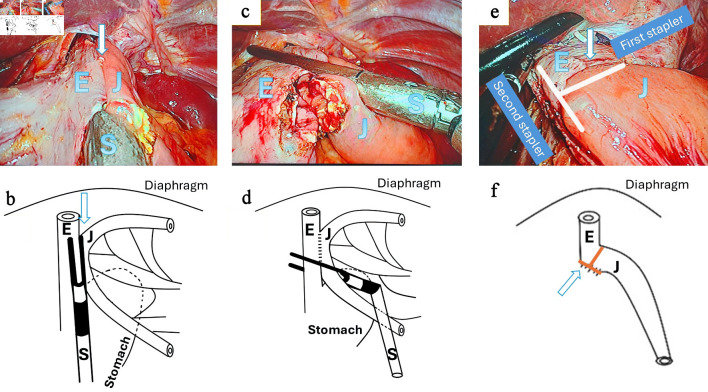
Intracorporeal T-shaped functional end-to-end esophagojejunostomy. (a, b) Creation of the initial side-to-side esophagojejunostomy. Small enterotomies are made on the lateral wall of the esophagus (E) and the antimesenteric border of the jejunum (J), followed by insertion and firing of a linear stapler (S) to create a functional side-to-side anastomosis (arrow). (c, d) Perpendicular stapling step. A second linear stapler is applied perpendicular to the initial staple line, incorporating both the esophageal and jejunal walls at the level of the common entry hole. Upon firing, this step closes the common entry hole and trims the overlapping tissue edges rather than resecting the anastomosis. (e, f) Final configuration of the T-shaped functional end-to-end esophagojejunostomy. The intersection of the two staple lines creates a characteristic T-shaped geometry (arrow), resulting in a straight luminal alignment without a residual blind pouch or jejunal stump. The apparent stump-like structure corresponds to the transverse staple line rather than a true jejunal stump. E: esophagus; J: jejunum; S: stapler.

**Figure 2:**
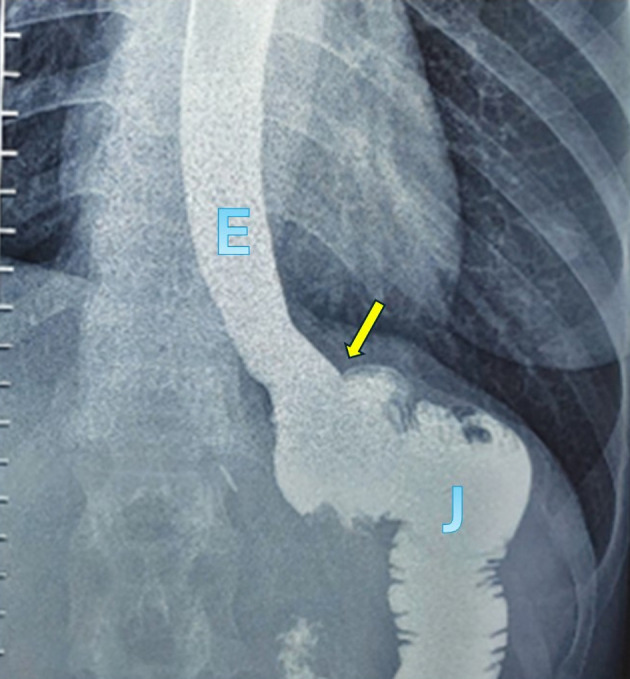
Postoperative contrast study. Contrast study shows a smooth and continuous luminal passage from the esophagus (E) to the jejunum (J), without evidence of a blind pouch or diverticular formation. The arrow indicates the anastomotic site.

Reconstruction was performed using a modified intracorporeal T-shaped functional end-to-end esophagojejunostomy. A jejunal limb was prepared 40–60 cm distal to the ligament of Treitz. Small enterotomies were created on the antimesenteric border of the jejunum and the lateral wall of the esophagus. Through these openings, an initial side-to-side esophagojejunostomy was constructed using a linear stapler (Fig. 1a, b). Subsequently, a second linear stapler was applied perpendicular to the initial staple line, incorporating the adjacent walls of both the esophagus and jejunum at the level of the common entry hole. Upon firing, this stapling step simultaneously closed the common entry hole and trimmed the overlapping tissue edges, rather than resecting the anastomotic site (Fig. 1c, d). This maneuver eliminated any potential blind pouch and reshaped the anastomosis into a straight luminal alignment. The intersection of the two staple lines resulted in a characteristic T-shaped configuration, corresponding to a functional end-to-end anastomosis (Fig. 1e, f). No jejunal stump was involved in the anastomosis (Fig. 2).

The duodenum was transected using a linear stapler 1–1.5 cm distal to the pylorus. Reconstruction was completed using a Roux-en-Y configuration, with an intracorporeal side-to-side jejunojejunostomy performed using a linear stapler to restore intestinal continuity. Abdominal drains were placed selectively, and specimens were retrieved in a protective bag through an enlarged port site.

### Perioperative management

Perioperative care followed standardized enhanced recovery principles, including early mobilization, early oral intake as tolerated, and selective use of nasojejunal decompression. Postoperative complications were recorded and graded according to the Clavien–Dindo classification.

### Outcome measures

The primary outcomes were technical feasibility of intracorporeal T-shaped esophagojejunostomy and anastomotic-related complications, including leakage, stricture, and anastomotic recurrence. Secondary outcomes included operative time, intraoperative blood loss, lymph node yield, postoperative recovery parameters (time to first flatus, time to oral intake, and length of hospital stay), and long-term oncologic outcomes, including overall survival (OS) and recurrence patterns.

### Follow-up

Patients were followed according to institutional protocols, with clinical assessment, laboratory testing, and imaging at regular intervals. Survival status and recurrence data were obtained from outpatient visits and medical records. Follow-up was complete for all patients.

### Statistical analysis

Continuous variables are presented as mean ± standard deviation or median with range, as appropriate. Categorical variables are expressed as frequencies and percentages. Overall survival (OS) was estimated using the Kaplan–Meier method. Statistical analyses were performed using SPSS software (version 26; IBM Corp., Armonk, NY, USA).

### Data availability

All relevant data are included within the manuscript and its supporting information files. Deidentified individual-level data are available from the corresponding author upon reasonable request.

## Results

### Patient characteristics

Between July 2017 and July 2025, 167 consecutive patients with gastric cancer underwent TLTG with D2 LND and intracorporeal T-shaped functional end-to-end esophagojejunostomy. The cohort predominantly consisted of patients with locally advanced disease (stage II–III: 77.3%), reflecting routine clinical practice in a tertiary referral center. Baseline characteristics are summarized in Table 1.

**Table 1:** Baseline Characteristics of the Study Population (N = 167)

Age (years)	60.97 ± 12.05 (range: 26–88)
BMI (kg/m^2^)	18.07 ± 6.40
Sex	
Male	118 (70.7%)
Female	49 (29.3%)
ASA physical status	
I	71 (42.5%)
II	73 (43.7%)
III	23 (13.8%)
Tumor location	
Upper third	20 (12.0%)
Middle third	141 (84.4%)
Diffuse infiltrative	6 (3.6%)
Histological type	
Tubular or papillary adenocarcinoma	99 (59.3%)
Mucinous adenocarcinoma	36 (21.5%)
Signet-ring cell carcinoma	32 (19.2%)
Histological differentiation	
Well differentiated	38 (22.8%)
Moderately differentiated	51 (30.5%)
Poorly/undifferentiated	78 (46.7%)
Pathological stage (JGCA)	
IA	18 (10.8%)
IB	20 (12.0%)
IIA	43 (25.7%)
IIB	33 (19.8%)
IIIA	14 (8.4%)
IIIB	31 (18.6%)
IIIC	8 (4.8%)

Values are presented as mean ± standard deviation or n (%), unless otherwise indicated. Pathological stage was defined according to the Japanese Gastric Cancer Association (JGCA) Classification, Fifth Edition. ASA: American Society of Anesthesiologists physical status classification.

The mean age was 60.9 ± 12.1 years (range: 26–88), and 118 patients (70.7%) were male. Most patients were classified as American Society of Anesthesiologists (ASA) I–II (86.2%). Tumors were predominantly located in the middle third of the stomach (84.4%), followed by the upper third (12.0%), while 3.6% presented with diffuse infiltrative type.

### Operative outcomes

All procedures were completed entirely laparoscopically without conversion to open surgery, and D2 LND was achieved in all patients. Intracorporeal T-shaped functional end-to-end esophagojejunostomy was successfully performed in 100% of cases, demonstrating the technical feasibility of the procedure (Table 2). In the early phase of our experience, a nasojejunal tube was routinely placed to allow decompression and to monitor potential anastomotic bleeding. As our experience with the T-shaped anastomosis increased and its safety became evident, we progressively reduced the use and duration of tube placement. In this series, 105 patients (62.9%) did not undergo postoperative nasojejunal tube placement (Table 2).

**Table 2:** Operative Outcomes (N = 167)

Technical feasibility	
Estimated blood loss (mL)	34.04 ± 14.30
Conversion to open surgery	0 (0%)
Nasojejunal tube placement	62 (37.2%)
Operative time (min)	207.01 ± 36.0 (range: 120–300)
Oncologic quality	
Retrieved lymph nodes (n)	22.95 ± 6.45 (range: 15–45)
Metastatic lymph nodes (n)	2.40 ± 2.75 (range: 0–12)
Proximal resection margin (cm)	5.2 ± 0.9 (range: 2.5–7.0)
Negative proximal and distal margins	167 (100%)
Intraoperative complications	
Splenic vessel injury	3 (1.8%)
Splenic parenchymal injury	4 (2.4%)
Hepatic parenchymal injury	3 (1.8%)
Middle colic artery injury	1 (0.6%)
Small bowel serosal injury	1 (0.6%)
Technical errors during anastomosis	3 (1.8%)

Values are presented as mean ± standard deviation or n (%), unless otherwise indicated. Estimated blood loss was calculated intraoperatively by the anesthesiology team. Technical errors during anastomosis were defined as intraoperative stapler misfiring or need for additional suturing without clinical sequelae. D2 lymphadenectomy was performed according to the Japanese Gastric Cancer Association guidelines.

Perioperative safety was high, with minimal blood loss and acceptable operative time (207.0 ± 36.0 min). Importantly, no anastomotic leakage occurred, and no patient required reoperation for anastomotic failure (Table 2). Intraoperative adverse events occurred in 12 patients (7.2%), including splenic vessel or parenchymal injury (4.2%), hepatic injury (1.8%), colonic vessel injury (0.6%), and small bowel serosal injury (0.6%). Technical errors related to anastomosis occurred in three patients (1.8%), all of which were managed laparoscopically without conversion (Table 2).

### Postoperative outcomes

Overall postoperative morbidity was low (2.4%), and no postoperative mortality was observed. The absence of anastomotic leakage was consistent with a smooth postoperative course, allowing early recovery and discharge (Table 3).

**Table 3:** Postoperative and Long-Term Outcomes (N = 167)

Postoperative complications	
Pneumonia	1 (0.6%)
Residual intra-abdominal abscess	1 (0.6%)
Surgical site infection	2 (1.2%)
Anastomotic leakage	0 (0%)
Postoperative pain (VAS)	
Mild	77 (44.5%)
Moderate	72 (41.6%)
Severe	18 (10.4%)
Very severe	0 (0.0%)
Postoperative recovery	
Duration of nasojejunal tube placement (h)	92.43 ± 55.6 (range: 24–360)
Time to drain removal (days)	3.7 ± 1.6 (range: 2–7)
Time to first flatus (h)	49.58 ± 16.7 (range: 24–96)
Time to oral intake (days)	3.01 ± 1.09 (range: 2–6)
Postoperative hospital stay (days)	7.45 ± 1.89 (range: 5–15)
Adjuvant chemotherapy	
Yes	129 (77.2%)
No	38 (22.8%)
Long-term outcomes	
Mean follow-up duration (months)	44.10 ± 20.01 (range: 2–84)
Postoperative reflux symptoms	33 (19.8%)
Anastomotic stricture	3 (1.8%)
Postoperative bowel obstruction	2 (1.8%)
Anastomotic recurrence	1 (0.6%)
Distant metastasis	31 (18.6%)
Time to metastasis (months)	22.5 ± 9.1 (range: 8–52)
Mean overall survival (months)	54.38 ± 1.98 (range: 31.15–56.85)

Values are presented as mean ± standard deviation or n (%), unless otherwise indicated. Postoperative complications were defined as events occurring within 30 days after surgery. Pain intensity was assessed using the visual analog scale (VAS). Overall survival was estimated using the Kaplan–Meier method.

Postoperative recovery was rapid, with early return of bowel function, early resumption of oral intake, and a short hospital stay. Routine nasojejunal decompression was omitted in most patients (62.9%) without an increase in anastomotic complications, supporting the feasibility of an enhanced recovery pathway after TLTG (Table 3).

### Long-term outcomes

Follow-up was complete for all patients, with a mean duration of 44.1 ± 20.0 months. Late anastomotic complications were infrequent, with anastomotic stricture occurring in only 1.8% of patients, all managed conservatively, and only one case of anastomotic recurrence observed. Distant metastases developed in 18.6% of patients, with a mean time to metastasis of 22.5 months. The mean OS was 54.4 months. (Table 3).

## Discussion

TLTG has been increasingly adopted as a standard minimally invasive approach for gastric cancer, particularly in East Asia, owing to its advantages in postoperative recovery and comparable oncologic outcomes to open surgery [2–4]. Nevertheless, intracorporeal esophagojejunostomy remains one of the most technically challenging steps of the procedure, and an optimal reconstructive method has not yet been universally accepted [5].

In the present study, the absence of anastomotic leakage in a relatively large cohort represents a notable finding, given that leakage remains one of the most critical complications after total gastrectomy [6, 7]. From a technical standpoint, this may be explained by the geometric configuration of the anastomosis. The perpendicular stapling step not only closes the common entry hole but also removes the lateral component of the initial side-to-side construction, resulting in a straight luminal axis without a residual blind pouch. This configuration may reduce local stasis, improve luminal flow, and decrease mechanical stress at the anastomotic site, which are factors potentially associated with improved anastomotic integrity. Accordingly, this technique may represent a technically meaningful modification of functional end-to-end anastomosis, in which geometric standardization of the anastomotic configuration contributes to reproducibility while maintaining favorable clinical outcomes.

Compared with established techniques, the present method demonstrates several conceptual differences. Conventional overlap anastomosis is fundamentally side-to-side and may result in a residual blind pouch, which has been associated with delayed emptying or food stasis. In contrast, functional end-to-end anastomosis aims to create a straight luminal continuity, although its configuration may vary depending on the closure technique [5–7]. The T-shaped modification in this study provides a more defined geometric transition by using a perpendicular stapling maneuver, creating two intersecting staple lines that standardize the final configuration. This may contribute to greater reproducibility, particularly in totally laparoscopic settings where instrument maneuverability is limited. Circular stapler techniques, while widely used, may be technically demanding in intracorporeal reconstruction and dependent on additional devices or steps [5, 7].

From a clinical perspective, the low rate of anastomosis-related complications observed in this study is consistent with the proposed technical advantages. Although a direct comparison was not performed, the outcomes appear comparable to, and in some aspects favorable to, previously reported laparoscopic series [2–4, 9]. In addition, the omission of routine nasojejunal tube placement did not result in increased complications, suggesting that the anastomotic configuration may be sufficiently stable to support early postoperative recovery pathways.

While this study primarily focuses on technical aspects, the observed long-term outcomes, including overall survival and recurrence patterns, were comparable to those reported in other laparoscopic gastrectomy series [2–4, 9]. These findings suggest that the modified reconstruction does not compromise oncologic principles when standard surgical requirements, including adequate proximal margins and D2 lymphadenectomy, are fulfilled [8].

Nevertheless, the favorable outcomes observed in this study may be influenced by surgical expertise, as all procedures were performed in a high-volume setting. It is likely that both technical design and operator experience contribute to the results. However, the structured sequence and consistent geometry of the T-shaped configuration may facilitate learning and improve reproducibility once the learning curve is achieved.

This study has limitations inherent to its observational non-comparative design, which precludes direct comparison with other anastomotic techniques. Additionally, as this study was conducted in high-volume centers, the generalizability of the results to low-volume institutions may be limited. Further prospective comparative or propensity-matched studies are required to confirm the advantages of this technique over existing methods.

### Conclusions

TLTG with D2 LND and intracorporeal T-shaped functional end-to-end esophagojejunostomy using a linear stapler is a safe, feasible, and reproducible technique for the treatment of gastric cancer. This approach is associated with low anastomotic-related complications, acceptable postoperative morbidity, and favorable long-term oncologic outcomes. The results support its use as a reliable reconstructive option following TLTG in appropriately selected patients.

## Data Availability

The datasets generated and analyzed during the current study are available from the corresponding author upon reasonable request.

## Abbreviations

ASA: American Society of Anesthesiologists
D2 LND: D2 lymphadenectomy
JGCA: Japanese Gastric Cancer Association
OS: overall survival
STROBE: Strengthening the Reporting of Observational Studies in Epidemiology
TLTG: totally laparoscopic total gastrectomy
VAS: visual analog scale
